# Patterns of structural and sequence variation within isotype lineages of the *Neisseria meningitidis* transferrin receptor system

**DOI:** 10.1002/mbo3.254

**Published:** 2015-03-19

**Authors:** Paul Adamiak, Charles Calmettes, Trevor F Moraes, Anthony B Schryvers

**Affiliations:** 1 Department of Microbiology, Immunology and Infectious Diseases, University of CalgaryCalgary, Alberta, T2N 4N1, Canada; 2 Department of Biochemistry, University of TorontoToronto, Ontario, M5S 1A8, Canada

**Keywords:** Iron acquisition, outer membrane protein, sequence variation, transferrin receptor

## Abstract

*Neisseria meningitidis* inhabits the human upper respiratory tract and is an important cause of sepsis and meningitis. A surface receptor comprised of transferrin-binding proteins A and B (TbpA and TbpB), is responsible for acquiring iron from host transferrin. Sequence and immunological diversity divides TbpBs into two distinct lineages; isotype I and isotype II. Two representative isotype I and II strains, B16B6 and M982, differ in their dependence on TbpB for in vitro growth on exogenous transferrin. The crystal structure of TbpB and a structural model for TbpA from the representative isotype I *N. meningitidis* strain B16B6 were obtained. The structures were integrated with a comprehensive analysis of the sequence diversity of these proteins to probe for potential functional differences. A distinct isotype I TbpA was identified that co-varied with TbpB and lacked sequence in the region for the loop 3 *α*-helix that is proposed to be involved in iron removal from transferrin. The tightly associated isotype I TbpBs had a distinct anchor peptide region, a distinct, smaller linker region between the lobes and lacked the large loops in the isotype II C-lobe. Sequences of the intact TbpB, the TbpB N-lobe, the TbpB C-lobe, and TbpA were subjected to phylogenetic analyses. The phylogenetic clustering of TbpA and the TbpB C-lobe were similar with two main branches comprising the isotype 1 and isotype 2 TbpBs, possibly suggesting an association between TbpA and the TbpB C-lobe. The intact TbpB and TbpB N-lobe had 4 main branches, one consisting of the isotype 1 TbpBs. One isotype 2 TbpB cluster appeared to consist of isotype 1 N-lobe sequences and isotype 2 C-lobe sequences, indicating the swapping of N-lobes and C-lobes. Our findings should inform future studies on the interaction between TbpB and TbpA and the process of iron acquisition.

## Introduction

*Neisseria meningitidis* is a Gram-negative diplococcal bacterium that is responsible for a substantial burden of global meningitis and bacteremia (Rosenstein et al. 2001; Stephens et al. 2007). In addition to its' ability to cause devastating invasive disease, *N. meningitidis* is also found as part of the commensal nasopharyngeal flora; present in ∼10–15% of healthy adults (Cartwright et al. 1987; Durey et al. 2012). Similar to other pathogenic and commensal organisms in this microenvironment, *N. meningitidis* is naturally competent and able to incorporate exogenous genetic material into its own genome but preferentially uptakes and incorporates DNA from related bacteria due to the presence of specific uptake sequences (Zhang et al. 2013). This enables rapid variation of specific genes and highly adaptive responses to host selective pressures. The patterns of genetic variations within specific genes and multigene systems resulting from these pressures provide insight into the functional and structural constraints on evolution within these types of systems (Marks et al. 2012). This understanding is fundamental for the design and development of antigens with improved vaccine coverage.

*Neisseria meningitidis* is highly adapted to its human host and acquires necessary iron in the host environment using specialized receptor systems that sequester iron from circulating host glycoproteins; notably transferrin (Tf) (Schryvers and Morris 1988b). The transferrin receptor system consists of two component proteins, an integral membrane protein, transferrin-binding protein A (TbpA), and a lipoprotein, transferrin-binding *protein* B (TbpB) (Morgenthau et al. 2013). TbpA functions as a TonB-dependent conduit allowing iron transport through the outer membrane. TbpB extends from the outer membrane surface by virtue of an N-terminal peptide region that is anchored to the outer membrane by fatty acyl chains on the N-terminal cysteine and is involved in the initial capture of iron-loaded Tf.

The crystal structure of an *N. meningitidis* TbpA complexed with human Tf (hTf) revealed that the C-lobe of hTf was in a partially open conformation (Noinaj et al. 2012), indicating that binding process involved separation of the C1 and C2 domains that normally are in close juxtaposition in order to complete the co-ordination of ferric ion. The structure also revealed that an *α*-helix from TbpA was inserted between the C1 and C2 domains along with a loop from the N-terminal plug domain suggesting that it facilitates the removal of iron from hTf.

In contrast to the complex of TbpA and hTf, the C-lobe of hTf is in a fully closed conformation in the structure of a complex of TbpB and hTf (Calmettes et al. 2012). This explains the very strong preference for binding the iron-loaded form of hTf by TbpB, and since TbpB can extend substantially from the surface of the outer membrane, it is ideally suited for the initial capture of hTf. The observation that formation of a ternary complex of TbpA, TbpB and Tf requires the presence of a nearly intact anchor peptide (Yang et al. 2011) implicates regions of the anchor peptide and TbpA in formation of the ternary complex. Although speculative, the proposal that conformational changes in the anchor peptide induced by binding holo Tf facilitate formation of the complex with TbpA is an appealing hypothesis (Yang et al. 2011). However, the process by which the transfer of Tf to TbpA results in domain separation and in requisite modification of the TbpB-Tf interaction is not understood. Although structural models have been proposed for the ternary complex (Noinaj et al. 2012; Morgenthau et al. 2013) they do not provide sufficient insight to understand the process, thus high-resolution structural information and further experimental studies are required.

TbpB exhibits a high degree of genetic sequence variability between strains (Ferrón et al. 1992; Rokbi et al. 1997) and two distinct lineages of TbpB variants, isotype I and isotype II, have been described. The isotypes are unique from each other with respect to size and antigenic distinctiveness (Rokbi et al. 2000; Renauld-Mongenie et al. 2004). In addition, two strains representing the isotype 1 (B16B6) and isotype 2 (M982) lineage, differed in their ability to grow on exogenous hTf as an iron source in vitro when the *tbpB* gene was interrupted (Irwin et al. 1993; Renauld-Mongenie et al. 2004). TbpB was also not required for the in vitro growth of *N. gonorrheae* (Anderson et al. 1994), *Haemophilus influenza* (Gray-Owen et al. 1995), or *Moraxella catarrhalis* (Luke and Campagnari 1999). However, it has not been firmly established whether the dependence upon TbpB is a function of the genetic background or properties of either of the receptor proteins. It is also important to recognize that TbpB has been shown to be essential for survival and disease causation in a pig infection model in spite of the inability to demonstrate its requirement for growth in vitro (Baltes et al. 2002). Although there is a suggestion of functional differences between receptors from strains with isotype I and isotype II TbpBs, whether this is reflected by structural differences in the TbpBs or TbpAs is currently unknown. The current study was initiated to explore the sequence and structural diversity of the TbpB and TbpA proteins to gain insights into the covariation and coevolutionary correlations between the TbpA proteins partnered with each TbpB isotype.

## Experimental Procedures

### Transferrin receptor sequence sources

*Neisseria meningitidis* transferrin receptor sequences for this study were acquired from two independent sources. The first were sequences we obtained from a locally maintained, historical, global strain collection (Table S3) provided by Dominique Caugant (Norway Institute of Public Health, Oslo, Norway). These samples are epidemiologically diverse with regard to bacterial strain type, year of isolation, and country of origin. This collection was used to ensure that a subset of both *tbpA* and *tbpB* genes could be acquired from the same isolate. The sequences were submitted to NCBI and the accession numbers are listed in Table S3. Additional sequences were obtained from the *Neisseria* Bacterial Isolate Genomic Sequence Database (BIGSDB) (http://pubmlst.org/neisseria/) (Jolley and Maiden 2010). The genes are numbered according to their numbering in the BIGSDB and accessed under the All loci tab, Other schemes, Iron acquisition heading. The final set consisted of 213 TbpA sequences, 80 from the strain collection and 133 from the BIGSDB, and 229 TbpB sequences, 69 from the strain collection and 160 from the BIGSDB.

### DNA extraction and sequencing from local strain collection

All isolates were grown on Brain Heart Infusion (BHI) agar overnight at 37°C in the presence of 5% CO_2_. DNA template was prepared in one of two ways. Bacteria were harvested *from* agar plates and resuspended in sterile phosphate buffered saline (PBS). The cells were centrifuged at 13,200*g* for 30 sec and the pellet re-suspended in fresh PBS to wash. Cells were either heat killed at 55°C for 1–2 h, or DNA was extracted using the DNAEasy kit (used according to the manufacturer's specifications, Qiagen, Gaithersburg, MD) and eluted in 200 *μ*L of sterile distilled water (SDW).

Polymerase chain reaction (PCR) was used to amplify matched *tbpA* and *tbpB* genes from the prepared genomic DNA. The PCR master mix consisted of 12 *μ*L of SDW, 2.5 *μ*L of PCR reaction buffer, 2.5 *μ*L of 2 mmol/L dNTPs, 2.5 *μ*L of each primer at 5 *μ*mol/L, 1.5 *μ*L of Taq polymerase (Thermo Scientific, Ottawa, ON, Canada), 0.5 *μ*L of pfu polymerase (Thermo Scientific), and 1 *μ*L of genomic DNA template. The primers used to amplify and sequence *tbpA* were designed as part of this investigation (Table S1A), and the primers used to amplify and sequence *tbpB* were adapted from a separate study (Table S1B) (Harrison et al. 2008). PCR to amplify *tbpA* was performed under the following cycling conditions using a BioRad Thermocycler (Mississauga, Ontario, Canada): preamplification denaturation at 94°C for 2 min; 35 cycles of 94°C for 30 sec, 60°C for 30 sec, 72°C for 3 min; final extension of 72°C for 5 min. The program to amplify *tbpB* was implemented as follows: 94°C for 2 min; 35 cycles of 94°C for 30 sec, 45°C for 30 sec, 72°C for 2 min; final extension of 72°C for 5 min. Sequencing was performed by Macrogen USA (Rockville, MD) or the University of Calgary DNA Services. Sequencing primers for each *tbpA* and *tbpB* are identified in Table S1.

### Inclusion Criteria from Neisseria Bacterial Isolate Genomic Sequence Database

BIGSDB maintain a collection of transferrin-binding protein A and transferrin-binding protein B genes from a variety of *Neisseria* spp. (Jolley and Maiden 2010). For this study *tbpA* and *tbpB* sequences were included from this database if they could be clearly identified as from *N. meningitidis* and were unique in the database.

### Expression and purification of recombinant *N. meningitidis* strain B16B6 TbpB

TbpB from *N. meningitidis* B16b6 was overexpressed in *Escherichia coli* Bl21 (DE3) as a maltose-binding protein fusion partner containing a polyhistidine tag and a TEV cleavage sequence site preceding the region encoding the mature TbpB_41-579_ protein. Cells were grown in 2YT medium at 37°C, and fusion protein expression was induced overnight at 20°C by addition of 0.5 mmol/L Isopropyl β-D-1-thiogalactopyranoside (IPTG). Cells were harvested at 5000*g* and resuspended in 100 mL of lysis buffer (25 mmol/L Hepes pH 7.5, 200 mmol/L NaCl) containing 1 mmol/L Phenylmethylsulfonyl fluoride (PMSF). Cells were lysed with two passes through a French Press cell and cell debris was removed by centrifugation at 18,000*g*. The supernatant fraction was loaded into a 5 mL HisTrap column GE healthcare, Pollards Wood, UK. The column was washed with 20 mmol/L imidazole in lysis buffer and the recombinant TbpB fusion protein was eluted with 250 mmol/L imidazole. The purified fusion protein was dialyzed for 18 h at room temperature (25 mmol/L Hepes pH 7.5, 20 mmol/L NaCl) in the presence of 1 mg of his-tagged TEV protease. Digested proteins were loaded onto a 5 mL HisTrap column: cleaved TbpB was collected in the flow-through and His-tagged MBP and TEV protease together with uncleaved fusion-proteins were collected together with 250 mmol/L imidazole. Pure TbpB was concentrated to 10 mg/mL using a centrifugal filter (cut off 50 kDa) then loaded into Superdex200 column (GE healthcare, Pollards Wood, UK) in order to check sample homogeneity.

### Protein crystallization

Purified TbpB was initially screened with 1:1 (protein:precipitant) ratio against the Index (Hampton research, Aliso Viejo, CA, USA), JSCG+ (Molecular Dimensions, Altamonte Springs, FL, USA), and the MCSG suite (Microlytics, Burlington, MA, USA) from commercial screen using sitting drop vapor diffusion at 8 and 13 mg/mL of TbpB_41-579_. Crystals were initially observed in 0.2 mol/L sodium chloride, 2.0 mol/L ammonium sulfate, 0.1 mol/L Bis-Tris at pH 6.5, then optimized with a 2:1 ratio hanging drop at 20°C in precipitant condition composed of 0.2 mol/L cesium chloride, 1.8 mol/L ammonium sulfate, 0.1 mol/L sodium citrate pH 5.8, and 15% (w/v) glycerol, yielding crystals in space group C2.

### Data collection and structure determination

Crystals were cryoprotected by transferring into Paratone-N oil and flash-cooled in liquid nitrogen. X-ray diffraction was collected on crystals frozen at 105 K on beamline 08B1-1 to the Canadian Macromolecular Crystallography Facility at the Canadian Light Source (CLS). Diffraction data set of B16B6 isotype I TbpB_41-579_ were collected at a wavelength of 2.16 Å using 1° oscillations with 360 images. Data were processed with XDS to resolution of 3.3 Å. The first structural model including the 3 copies of TbpB present in the asymmetric unit was successfully resolved by molecular replacement using PHASER. The molecular replacement search model was prepared using the M982 isotype II TbpB structure from the PDB entry 3VE2. For cross-validation, a random set of 5% of the totals reflections were kept aside from the refinement and used for the calculation of R_free_. The initial model was split in six groups consisting of each TbpB N- and C-lobes, which were refined as independent rigid bodies to position the three TbpB monomers. The final model was ultimately generated following several rounds of model building and refinement using Coot and PHENIX using noncrystallographic symmetry and TLS model, yielding a final Rwork/Rfree of 0.25/0.30. A Ramachandran plot calculated 13.6% of residues in the allowed region and 0.2% as outlier. The numbering of residues initiates with the first mature amino acid (Cis-1) remaining after signal peptide cleavage. Data collection and refinement statistics are indicated in Table S2.

### Structural analyses, multiple sequence alignments, and phylogenetic analysis

Protein DataBank (Berman et al. 2000) (pdb) files for the structures of the M982 TbpB (Calmettes et al. 2012) (PDBid 3VE2), the K454 TbpB (Noinaj et al. 2012) (PDBid 3V8U) and TbpA (Noinaj et al. 2012) (PDBid 3V8X) were acquired and visualized with the Pymol Software. Confirmatory analyses of secondary structure elements were conducted with the Dictionary of protein Secondary Structure Patterns (DSSP Joosten et al. 2011; Kabsch and Sander 1983). Multiple sequence alignments of all TbpA and TbpB variants were generated using the M-Coffee alignment algorithm as implemented on the T-Coffee server site (http://www.tcoffee.org/) (Moretti et al. 2007). Alignments were analyzed and edited using Geneious Pro (Drummond et al. 2011) and automatically cleaned using GBlocks (Castresana 2000).

Phylogentic trees were generated from cleaned alignments using the Maximum likelihood method, PhyML (Guindon et al. 2010), with 1000 Bootstraps, employing the general time reversible (GTR) model (Tavare 1986) (Dereeper et al. 2008, 2010). Normalized (0–100) BLOSUM62 conservation scores within alignments were calculated using the Molecbio Python package developed by Dave Curran (Curran 2012). Conservation mapped structures were produced by plotting the BLOSUM62 scores from the generated alignments against each amino acid position in the alignment. Alignment and phylogenetic tree annotations were performed using FigTree v1.4, MicroSoft Word 2008 (Microsoft Corp. Redmond, WA), MicroSoft Power Point 2008 and Adobe Illustrator CS6 (Adobe Systems, San Jose, CA).

## Results

The overall approach was to develop structural models for TbpB and TbpA from strain B16B6 to compare to known structures of Tbps from strains with isotype II TbpBs and use this insight to provide a structural context for exploring the sequence diversity of the receptor from of a large collection of *N. meningitidis* isolates. This approach was adopted to provide potential insights into structural constraints that might underpin the phylogenetic groupings, to explore the functional differences observed between strains B16B6 and M982, and to probe associations between TbpB and TbpA.

### TbpB structural variation

**Figure 1:**
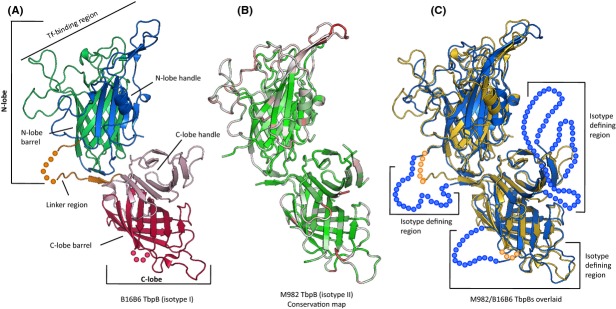
Panel A illustrates the *Neisseria meningitidis* TbpB crystal structure from isotype I strain B16B6. Green and red regions identify barrel domains of the N-lobe and C-lobe, respectively. Blue and pink identifies the handle domains. The linker region between lobes is colored orange. Each individual circle identifies an amino acid present in the protein but not resolved in crystal structure. Panel B indicates normalized Blosum 62 conservation scores mapped on to the annotated strain M982 *N. meningitidis* TbpB structure. Red colored regions correspond to a low conservation score (<33.33), white regions depict a moderate conservation score (33.33< and <66.66), and green regions identify high conservation scores (>66.66). Panel C presents the overlaid representative TbpB structures from M982 (Blue color, PDB ID: 3VE2) and B16B6 strains (colored in yellow) with key regions annotated. Isotype defining regions identify the features that differ consistently between isotype I and isotype II TbpBs. Each orange circle identifies a B16B6 amino acid that was not resolved in the crystal structure. The blue circles identify individual amino acids not resolved in the M982 istoype II structure.

Structures are available for two isotype II TbpBs from strains M982 (PDB ID: 3VE2) (Calmettes et al. 2012) and K454 (PDB ID: 3V8U) (Noinaj et al. 2012). These two istoype II TbpB structures are nearly identical, sharing 83.5% sequence identity and a RMS of 0.43 (Fig. S1). We solved the structure of the isotype I TbpB from strain B16B6 (Fig.1A, PDB ID: 4QQ1) in order to compare it to the known isotype II structures (Fig.1B). The overall structural organization was similar, consisting of two orthogonally oriented lobes comprised of N-terminal handle and C-terminal barrel domains. In contrast to the isotype II TbpBs, the loop regions of the handle and barrel domains of the N-terminal lobe were fully resolved in the B16B6 TbpB structure. Similarly, there was only one unresolved loop region in the C-lobe of the B16B6 TbpB compared to four in the type II TbpB structures (Fig.1C). Although much of the core secondary structural elements of both isotypes are nearly superimposable, there are subtle differences in loop regions and a tilt of a helix in the N-lobe handle domain (Fig.1C – extended description in Supplementary Section).

The primary differences between the TbpB isotypes are present in the unresolved regions of the crystal structure where there is poor electron density in the maps found in the linker region between lobes, and loop regions of the C-lobe (Fig.1C, dotted regions – extended description in Supplementary Section). The residues comprising the anchoring peptide that attaches to the outer membrane are not resolved in any of the structures (Fig.1 and Fig. S1). As such, important differences in these regions cannot be described from the X-ray-derived structural data alone, and differences in the amount of structure present in the models may influence the RMS calculation.

### TbpA structural variation

**Figure 2:**
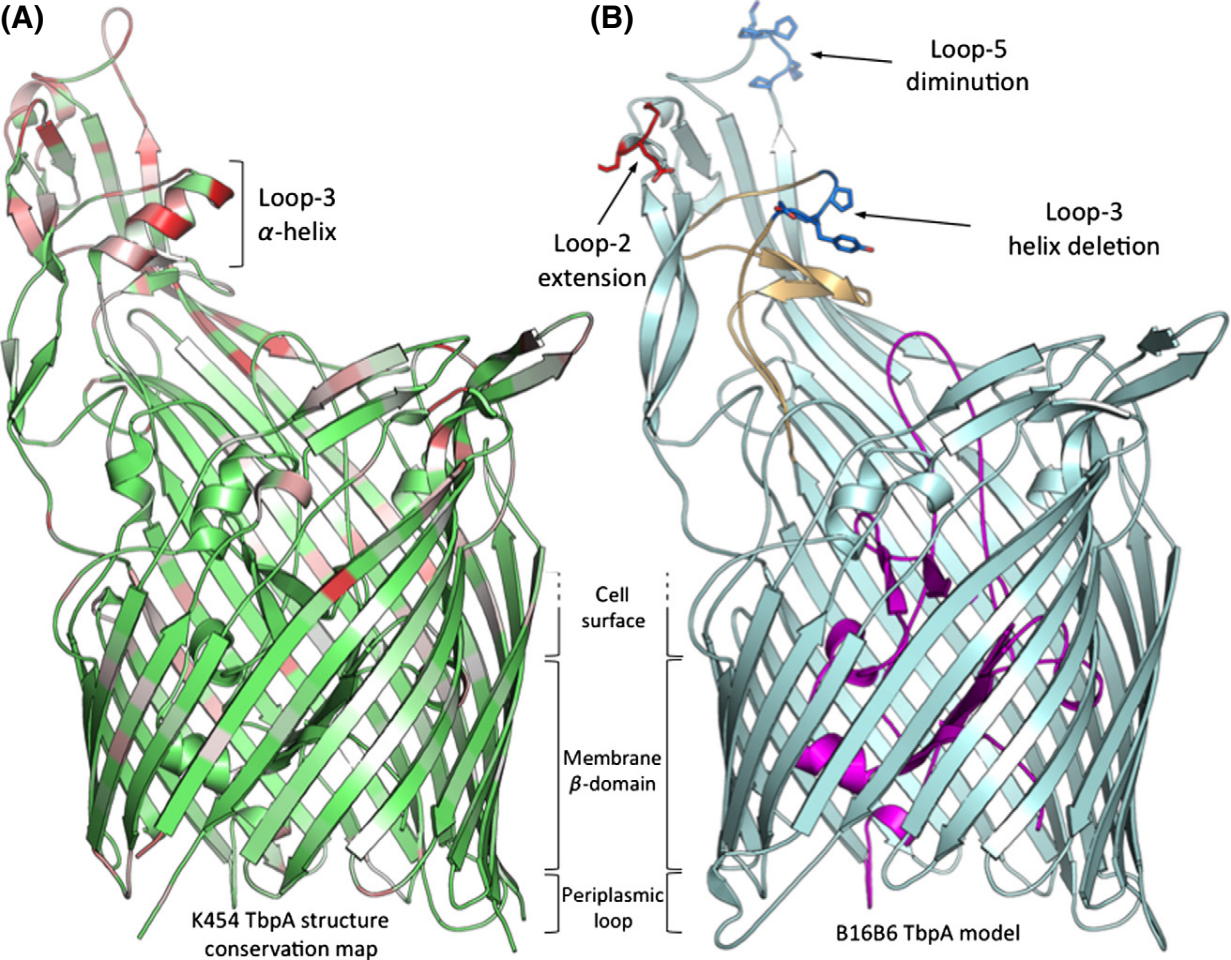
Cartoon of Structural Model of TbpA from Strain B16B6. Panel A illustrates normalized Blosum 62 conservation scores mapped on to the annotated strain K454 *Neisseria meningitidis* TbpA structure. Red colored regions correspond to a low conservation score (<33.33), white regions depict a moderate conservation score (33.33< and <66.66), and green regions identify high conservation scores (>66.66). Structural model of the B16B6 TbpA model is presented in panel B; the Nterminal plug region is colored in magenta and the C-terminal barrel region is colored cyan. The region in loop-2 where the B16B6 TbpA is larger than isotype I TbpA is highlighted in red stick, whereas the regions in loop-3 and loop-5 where there are gaps in the B16B6 sequence are indicated in blue. The loop-3 is colored in orange.

The published structure of a complex of hTf or hTf C-lobe with TbpA from *N. meningitidis* strain K454, (Noinaj et al. 2012) are the only structural models of TbpA available. The K54 model (Fig.2A) is a good representation for the M982 TbpA as there is relatively high sequence identity between the two TbpAs (95% identity). Since they have the identical number of amino acids the only anticipated structural differences are in the amino acid side chains. Although there is considerably less identity between B16B6 and K454 TbpA (75% identity), the overall structure is predicted to be very similar, with the greatest differences in areas where there are insertions or deletions relative to the K454 TbpA (Fig.2B). Of particular significance is the gap in loop-3 that overlaps the *α*-helix region in K454 that inserts between the domains of the hTf C-lobe. In this particular structural model the helix is absent. Although we cannot exclude the possibility that an alternate *α*-helix could be generated from an adjacent region of loop-3, structural modeling alone will not provide a definitive answer.

### TbpB sequence variation and phylogenetics

**Figure 3:**
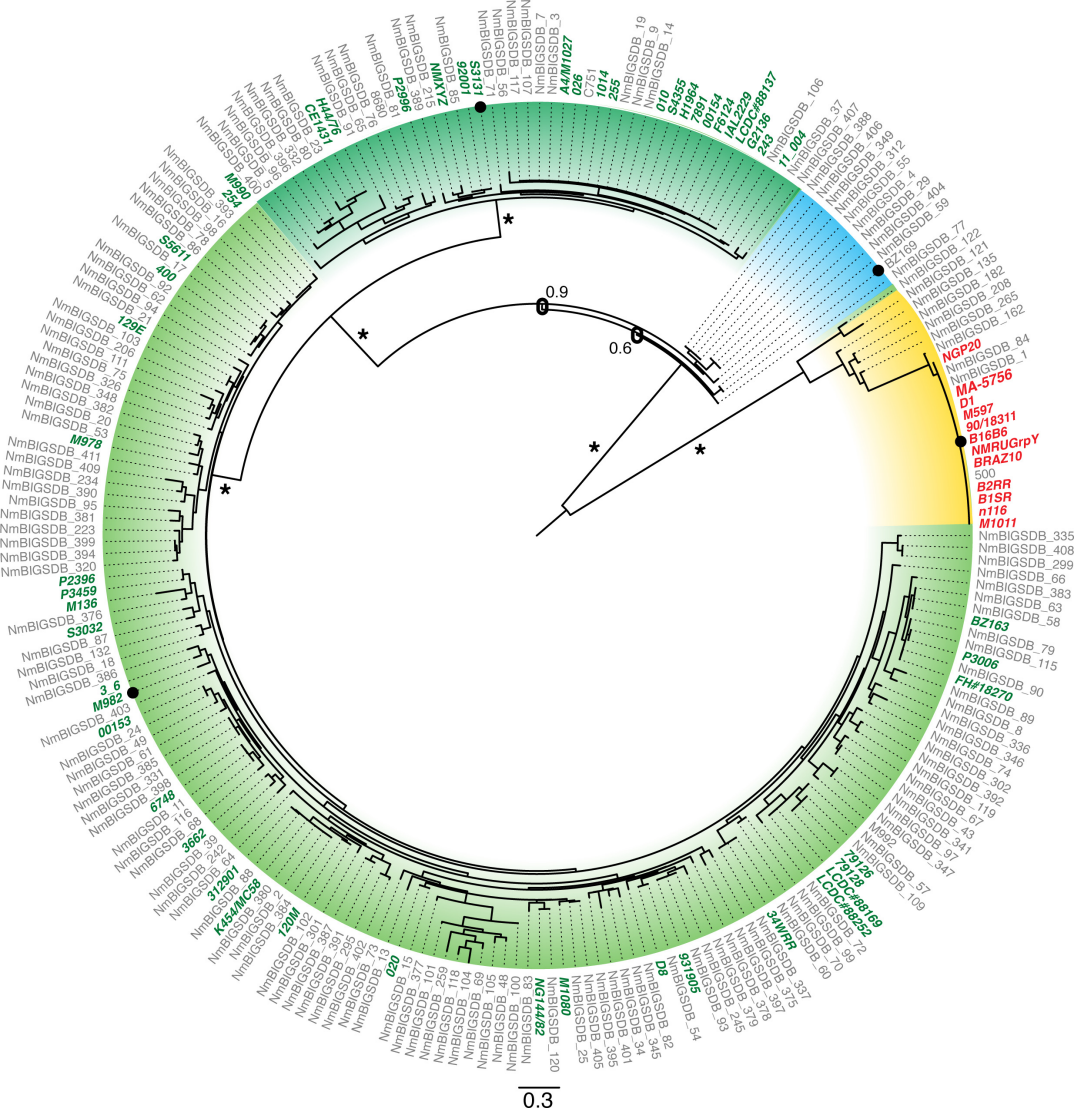
Maximum likelihood phylogenetic tree of the *Neisseria meningitidis* tbpB gene family. Leaf labels identify strains from which each of the 229 tbpB sequences was obtained. Two primary clades are identified within this tree corresponding to the isotype I and isotype II tbpB lineages (yellow background and green/blue backgrounds respectively). Support values for primary branches are depicted, and a “*” identifies branches with 100% support. Green and red leaf labels identify 52 isotype II and 12 isotype I strains from which the matching tbpA genes were obtained. Amino acid sequences for the leaves marked with a black circle are depicted in Figure4.

**Figure 4:**
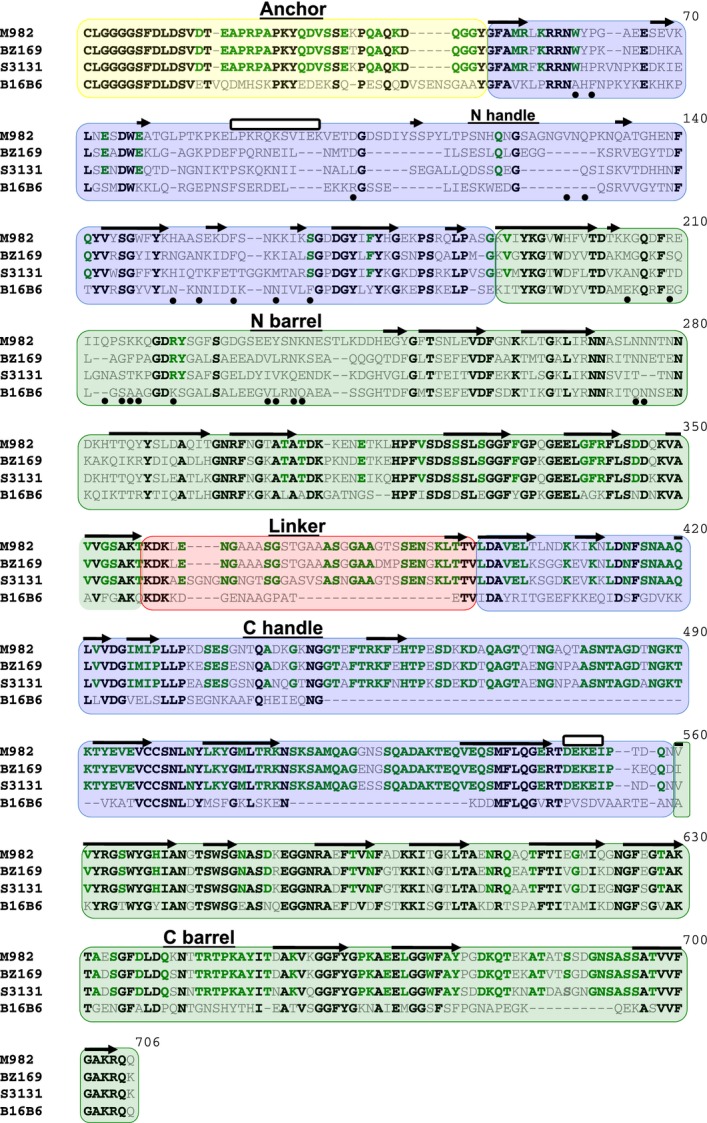
Representative TbpB amino acid sequences from strains M982, S3131, B16B6 and BZ169 (relative phylogenetic relationship identified in Fig.4). Green background identifies the barrel domains, blue background identifies the handle domains, yellow background identifies the anchor peptide, and red background identifies the linker region. Black colored sequence depicts conservation between all three representatives, green shows conservation within only isotype II strains, and gray shows no conservation. The black arrows represent β-strands, the white bars represent α-helices, and the black circles identify residues involved in the interaction between TbpB and human transferrin.

In order to obtain a comprehensive evaluation of the sequence diversity of TbpB we accessed the extensive collection of sequences available in public databases but also sequenced the *tbpB* and *tbpA* genes from a collection of *N. meningitidis* isolates to evaluate the covariation of TbpA with TbpB. Phylogenetic analysis identified four distinct branches (Fig.3), three isotype II branches (blue to green) and one corresponding to the isotype I variants (yellow). The four identified clades are well supported by boot strapping scores, however, within each clade the support values vary greatly (0–100% branch support). As a result no conclusions can be reliably drawn regarding trends for subgroups within the four clades. A sequence alignment prepared with TbpBs from each of the four major clades (Fig.4) illustrates the overall sequence diversity related to the key structural features of TbpB. As revealed by the structural study with the B16B6 TbpB, the most distinct differences between the isotype I and isotype II TbpBs is in the linker region and the C-lobe region where the large loops are reduced or absent in the isotype I TbpB. The alignment also reveals two distinct anchor peptide regions in the two isotypes.

Inspection of the alignment also reveals that the large loop regions in the C-lobe of isotype II TbpBs are fairly conserved, demonstrating that although there may be considerable overall sequence diversity in the C-lobe region, the variation within the isotypes is more limited. These observations are supported by phylogenetic analysis with C-lobe sequences (Fig. S2) demonstrating that there are only two distinct clusters representing the isotype I and isotype II TbpBs.

The sequence alignment illustrates that the major sequence diversity among all TbpBs resides in the loop regions of the N-lobe, particularly in the handle domain. A phylogenetic analysis with the N-lobe (Fig. S3) illustrates that this sequence diversity is primarily responsible for defining the four major clades of TbpBs. However, in this tree one of the isotype II clusters (blue color) identified from the intact *tbpB* tree branches with the isotype I group. This suggests that based on sequence alone, intact *tbpBs* within this cluster are isotype II, but the N-lobe sequence on its own shares more similarity to isotype I TbpBs.

Amongst the 23 N-lobe TbpB residues previously identified to interact with human transferrin (Calmettes et al. 2012), none are completely conserved amongst all TbpBs (Fig.4). This raises the question whether the binding residues might be relatively conserved within the individual clusters, so that exchanges within the N-lobe region may be tolerated within clusters, but could lead to nonfunctional proteins if exchanges occur between clusters.

### TbpA phylogenetics and sequence variation

**Figure 5:**
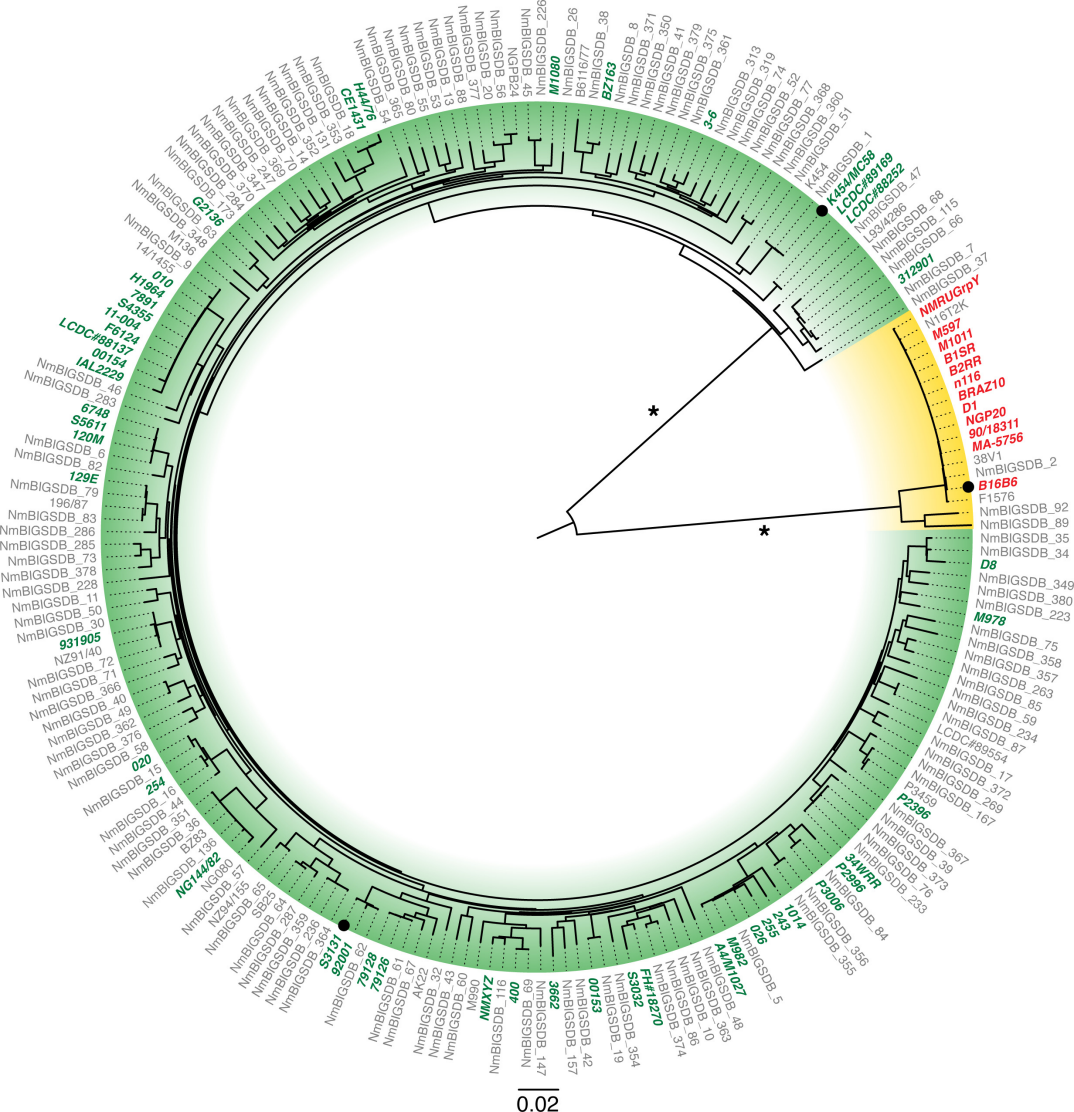
Maximum likelihood phylogenetic tree of the *Neisseria meningitidis* tbpA gene family. Leaf labels identify the 213 strains from which each of the tbpA sequences was obtained. Two primary clades are identified within this tree corresponding to the strains with isotype I and isotype II tbpB lineages. Support values for primary branches are depicted, and a “*” identifies branches with 100% support. Green and red labels identify 52 isotype II and 12 isotype I strains from which the matching tbpB genes were obtained. Amino acid sequences for the leaves marked with a black circle are depicted in Figure6.

As for TbpB, we accessed the extensive collection of TbpA sequences available in public databases that was supplemented with TbpA sequences from a collection of *N. meningitidis* isolates in order to provide paired TbpB sequences. Phylogenetic analysis of the TbpA sequences identified two distinct branches (Fig.5) whose members correspond perfectly to the isotype I and isotype II strains defined by the *tbpB* phylogenetics (100% branch support). No other correlations are readily observable amongst the other clusters within the *tbpA* groups.

**Figure 6:**
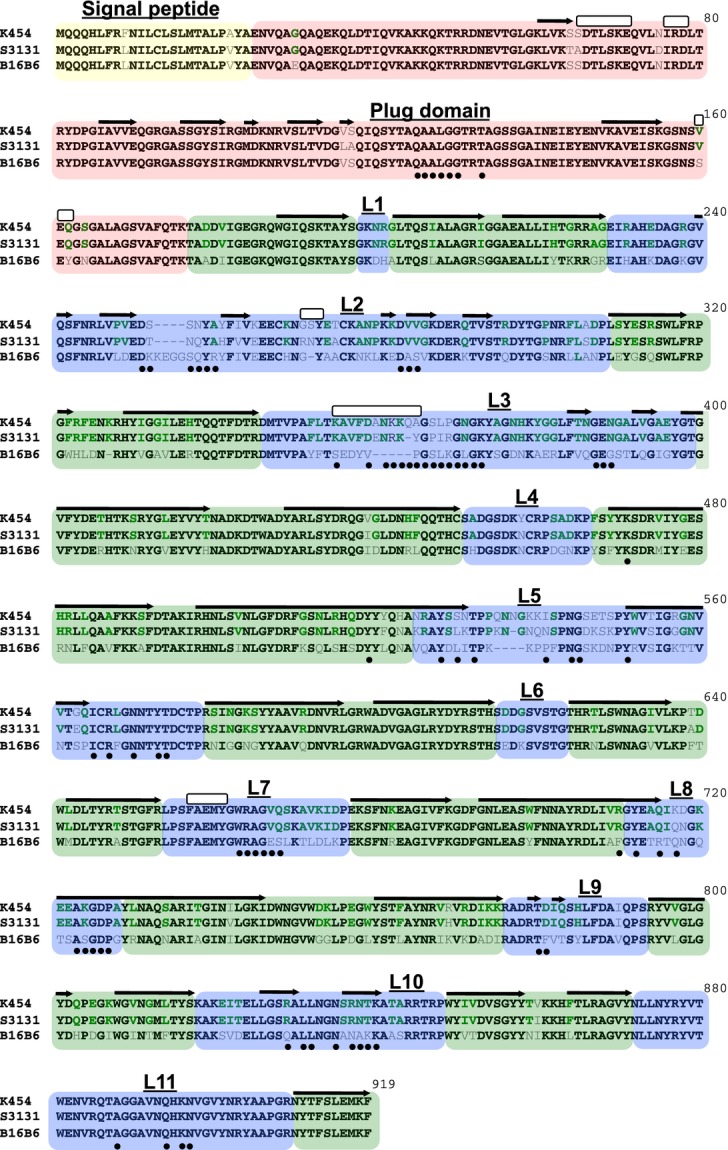
Representative TbpA amino acid sequences from strains K454, S3131 (isotype II strains) and B16B6 (isotype I strain). Green background regions correspond to the barrel and periplasmic components, blue background identifies extracellular loop, and the red background identifies the plug domain. Black colored sequence depicts conservation between all three representatives, green colored sequence depicts conservation within only the isotype II strains, and gray colored sequence identifies no conservation. The black arrows represent β-strands, the white bars represent α-helices, and the black circles identify residues involved in the interaction between K454 TbpA and human transferrin.

Three representative TbpAs (Fig.5) were selected for an alignment to illustrate the key features (Fig.6). It is apparent that the greatest sequence diversity is in the extracellular loop regions, particularly in the large extracellular loops 2, 3, and 5 (Figs.2, 6). Extracellular loops 3 and 5 are ∼5 amino acids longer in TbpAs from isotype II strains compared to those from isotype I strains (Fig.6). Additionally, the isotype I strain TbpAs lack ∼5 amino acids that make up the functionally important loop-3 *α*-helix. Of the 77 residues predicted to interact with Tf, only 37 are completely conserved within the TbpAs from this data set. The residues involved in the plug domain – Tf interaction are completely conserved in the full set, while the functional residues in loop-5 show a great deal of variability. Interestingly, the functional residues in the loop-3 *α*-helix are also not fully conserved amongst TbpAs from isotype II strains, and are largely absent in the TbpAs from isotype I strains (Fig.6).

### Variability within isotype I *Neisseria meningitidis* strains

In this analysis, the matched TbpAs and TbpBs from the isotype I strains each came from a single, multilocus sequence type (MLST) (Maiden et al. 1998), sequence type (ST) 11, of *N. meningitidis*. Within these strains, both transferrin-binding proteins (A and B) are completely conserved. This suggests that these strains might be direct clones, and that the consistent pairing of TbpA and TbpB isotypes is solely due to clonal descent within this group. To investigate this hypothesis, the strain's serotypes, and lactoferrin-binding protein (Schryvers and Morris 1988a) sequences were compared. The lactoferrin-binding proteins were selected as they share many similarities with the transferrin-binding proteins; both the transferrin and lactoferrin receptors consist of a transmembrane pore A protein, and a lipid anchored B protein, both are involved in iron transport, and importantly, both vary their sequences via similar mechanisms, under similar selective pressures (Beddek and Schryvers 2010; Adamiak et al. 2012).

For lactoferrin-binding protein A, sequences from isotype I strains: MA-5756, D1, B1SR, and 90/18311 were available at the time of this study. From lactoferrin-binding protein B, the sequences for isotype I strains: B16B6, B1SR, 90/*18311*, MA-5756, D1, BRAZ10 were available. The lactoferrin-binding protein B possesses a large hyper variable region that is proposed to mediate an important interaction with the cationic antimicrobial peptide, lactoferricin (Morgenthau et al. 2012). This section was removed for this analysis to prevent bias introduced by variability specific to this region. Within the sequences available, alignments of the lactoferrin-binding protein A revealed 100% conservation between different strains. However in the case of lactoferrin-binding protein B, only 69.1% of the sites were conserved. Additionally, the isotype I strains were typed as belonging to either serogroup B or serogroup C. These observations demonstrate that even though all of the isotype I TbpBs were found in ST11 *N. menigitidis* strains, these strains are not direct clones and there is evidence suggesting horizontal exchanges have occurred.

## Discussion

The observations in this study and prior studies demonstrate that the *N. meningitidis* transferrin receptor system exhibits a high degree of sequence variation that is likely due to a combination of efficient genetic exchange mechanisms and host evolutionary selective pressures (Hamilton and Dillard 2006). Uptake of exogenous DNA and homologous recombination provides a mechanism by which *N. meningitidis* can rapidly vary components under environmental selective pressures, which in turn is modulated by a novel CRISPR-mediated mechanism (Zhang et al. 2013). Given the immunogenic potential (Ala'Aldeen et al. 1994; Gorringe et al. 1995; Johnson et al. 1997), and surface exposure of TbpB (Calmettes et al. 2012) and parts of TbpA (Noinaj et al. 2012), it is reasonable to speculate that the observed variability has been driven by host immune selective pressures, and restrained by structural and functional requirements.

The pattern of TbpB sequence variation is counter intuitive to what one might expect, in that the functional (Tf binding) regions of the N-lobe are those that exhibit the most variation, while the C-lobe, whose function is not yet fully understood is much more highly conserved. The observation that mutation of individual N-lobe amino acids can have a dramatic impact on Tf binding yet the specific amino acids that bind Tf varies between TbpBs suggests that binding may be accomplished by different structural configurations in different TbpB lineages (Figs.4) (Moraes et al. 2009; Calmettes et al. 2011, 2012). This would predict that the variation in residues that directly bind Tf among diverse TbpBs may partially restrict the ready exchange of variable regions among TbpBs from different clusters or lineages as these might perturb the hTf interaction. Thus, although the overall diversity amongst the N-lobes may be great, its organization into clusters may make the challenge of antigenic variation for vaccine design more manageable.

Since the C-lobe is likely to be nearly as accessible at the cell surface as the N-lobe, the relative conservation of the C-lobe (Fig.4) is difficult to understand as it would be expected to be under similar pressures from the host immune system. It is possible that the function of TbpB involves as yet undiscovered interactions with other surface components that may be imposing constraints on the variation of the C-lobe.

TbpAs from different strains exhibit substantially less sequence and size variation than TbpBs and the observed variability is localized to the large, exposed loops that extend out beyond the membrane. Analogous to what is observed with TbpBs, most of these differences are located in regions shown to interact with Tf in the solved Tf – TbpA structure (Fig.6) (Noinaj et al. 2012). However, in contrast to TbpB (Calmettes et al. 2012), mutation of individual amino acids do not have a large impact on binding Tf (Noinaj et al. 2012), which suggests that there may be more tolerance to exchange of variants than with TbpB.

Prior studies identified two distinct lineages of meningococcal TbpBs based on size and antigenic differences (Rokbi et al. 1993, 2000; Renauld-Mongenie et al. 2004). One of the key differences between isotypes appears to be in the size of the C-lobe, whereby the istoype I TbpB possesses a substantially smaller C-lobe lacking the large flexible loops that were not resolved in the crystal structure of the isotype II TbpBs (Calmettes et al. 2012; Noinaj et al. 2012). Isotype I variants also possess longer anchor peptides and shorter linker regions than their isotype II counterparts. The observation that there are two distinct types of C-lobe and anchor peptide that distinguish the two TbpB isotypes (Fig.4 and Fig. S2) is perhaps not surprising in view of the intimate association between the anchor peptide and C-lobe (Moraes et al. 2009). The anchor peptide is observed in a locked conformation bound to the C-terminal amino acids of the C-lobe via an antiparallel B strand exchange, while the remaining anchor peptide wraps around the C-lobe domain.

Comparison of the *tbpA* and *tbpB* phylogenetic tree topology (Figs.5) demonstrates that those strains possessing isotype I TbpB variants also comprised a unique cluster of TbpA variants. The identification of an isotype I lineage of TbpA that is associated with the isotype I TbpB, and particularly the TbpB C-lobe (Fig. S2) begs the question of whether any TbpB-TbpA interaction could be responsible for this association. The observation that the anchor peptide is required for the formation of the ternary TbpA-TbpB-Tf complex (Yang et al. 2011), infers that there is an interaction between TbpA and the anchor peptide. This interaction is consistent with the association of a distinct type I TbpB anchor peptide (Fig.4) with the isotype I TbpA, even though the sites of interaction have not been identified. The intimate association of the anchor peptide and the C-lobe (Moraes et al. 2009) could in turn be partly responsible for the association between TbpA, the anchor peptide and C-lobe, although interactions with other factors not yet identified could be responsible.

Previous studies demonstrated that strain B16B6, which possesses an isotype I TbpB variant, was unable to grow with exogenous hTf as the sole source of iron when TbpB was not present (Irwin et al. 1993; Renauld-Mongenie et al. 2004). Under the same conditions, the isotype II strain M982 was able to grow without TbpB (Renauld-Mongenie et al. 2004). While this may be the result of other factors related to the strains genetic background, the results presented here suggest that differences in TbpA structure may explain this phenomenon. In particular, there is substantial variation in sequence and size of the region that encompasses the loop-3 *α*-helix that is proposed to be involved in triggering hTf to release iron (Noinaj et al. 2012). The reduced size and altered sequence of this region in isotype I strains suggests that the TbpA from isotype I strains employ a modified mechanism to accomplish the same task, or that it is in some way impaired relative to the TbpAs from isotype II strains. The demonstration that mutations of a *N. gonorrheae* TbpA enhanced the dependence upon TbpB for growth with exogenous Tf in vitro (Noto and Cornelissen 2008) may suggest that the isotype TbpA in strain B16B6 is similarly less efficient in acquiring iron.

The constraint that leads to co-variation of isotype I TbpB and TbpA appears to be unique to the transferrin receptor system, and is likely not due to a lack of opportunities for horizontal exchange, as strains possessing isotype I TbpB variants show evidence of horizontal exchange at both lactoferrin and capsular synthesis loci (Adamiak et al. 2012). The demonstration that the distinct isotype I and isotype II TbpB lineages extend to other *Neisseria* species (Harrison et al. 2008) raises the question as to whether the co-variation with TbpA is also maintained. It seems unlikely that selective pressures could maintain a lineage of TbpA that is less efficient in extraction of iron from Tf which begs the question of whether the lineage may have originally developed in the presence of a variant Tf for which it is more efficient in iron removal.

## Supporting Information

Table S1 (A) Transferrin binding protein A amplification and sequencing primers. (B) Transferrin binding protein B amplification and sequencing primers. **Table S2.** Data collection and refinement statistic for the *Nm* B16B6 TbpB crystal structure solved in this study. **Table S3.** Strain characteristics and accession numbers for TbpB and TbpA.

Figure S1 Superimposition of the N. *meningitidis* strain K454 (pink; PDB ID: 3V8U) and strain M982 (green PDB ID: 3VE2) TbpB crystal structures with key regions identified.

Figure S2 TbpB C lobe phylogenetic tree. TbpB C-lobe sequences from 229 strains were included in this analysis. Two primary clades are identified within this tree corresponding to the isotype I and isotype II *tbpB* lineages (yellow background and green backgrounds respectively). Support values for primary branches are depicted by a “*” identifying 100% support for these two branches.

Figure S3 TbpB N lobe phylogenetic tree. TbpB C-lobe sequences from 229 strains were included in this analysis. Two primary clades are identified within this tree corresponding to the isotype I and isotype II *tbpB* lineages (yellow background and green/blue backgrounds respectively). Support values for primary branches are depicted by a “*” identifying 100% support for these two branches.
