# Supplementary material for: Patterns of structural and sequence variation within isotype lineages of the Neisseria meningitidis transferrin receptor system
Source: Microbiologyopen. 2015 Mar 19;4(3):491–504. doi: 10.1002/mbo3.254 (PMC4475390; doi:10.1002/mbo3.254)
Supplement: Table S1 — (A) Transferrin binding protein A amplification and sequencing primers. (B) Transferrin binding protein B amplification and sequencing primers. Table S2. Data collection and refinement statistic for the Nm B16B6 TbpB crystal structure solved in this study. Table S3. Strain characteristics and accession numbers for TbpB and TbpA. [file mbo30004-0491-sd1.docx]

**Supplementary Section**

**Results: Description of comparison of isotype I and isotype II TbpB structures.**

Comparison of the B16B6 structure­­­ to the representative istoype II M982 structure (PDB ID: 3VE2) demonstrates that TbpBs from both isotypes share a very similar overall fold and organization (Figure 1C). The calculated N-lobe RMS is 1.9 angstroms using B16B6 N-lobe residues 43-328 aligned with M982 N-lobe residues 37-107, 121-264, 271-349. The calculated C-lobe RMS is 1.4 angstroms for B16B6 C-lobe residues 340-555, 560-576 aligned with M982 C-lobe residues 378-419, 475-499, 519-621, 627-660, 675-689. The overall overlay of the B16B6 and M982 entire proteins provides an RMS of 1.9 angstroms.

In each TbpB, the region above the N lobe barrel domain consists primarily of loops lacking secondary structural features except for a single small β-strand and two small α-helices. The N lobe handle domain consists of four prominent antiparallel β-strands and one large α-helix, with three smaller β-strands, and one smaller α-helix present in one of the loops. Similar to the N lobe barrel, the region extending from the surface of the C lobe barrel lacks secondary structural elements except for two small β-strands. The handle domains of the C lobes consist of eight antiparallel β-strands with unstructured loop regions between strands towards the N lobe handle, resembling the fHbp architecture, another Neisseriaceae surface exposed lipoprotein. Lastly, between each lobe there is a primarily unstructured linker region that contains only one clearly defined β-strand.

Only a portion of one C lobe barrel loop was unresolved for the B16B6 structure (4 residues between P555 and E560), but there are four unresolved loop regions in both of the istotype II structures including 77 and 89 residues missing for the TbpBs from the K454 and M982 strains, respectively (Figure 1 and Supplemental Figure 1). Similarly, substantial portions of the linker region between the two lobes are not resolved for any of the structures (6 unresolved amino acids in B16B6 TbpB’s linker, versus 23 and 29 missing residues within K454 and M982 TbpBs).

**Supplementry Table 1a. Transferrin binding protein A amplification and sequencing primers**

| Forward external primer | 5’ CGGCACCGCTCCTTCCGATGCCG 3’ |
| --- | --- |
| Reverse external primer | 5' GGAAATGAAGTTCTAAACGTCCAAACGCCGCAAATGCCGTCTGAAAGGCG  GATAACAATTTCACACAGG 3' |
| Forward internal sequencing primer isotype II | 5’ CGCATCGGCGGTGCGGAGG 3’ |
| Forward internal sequencing primer isotype I | 5’ CCTATTCGGGAAAAGACCATGCCC 3’ |
| Reverse internal sequencing primer | 5’ TCGCGATAGGCATTGTTG 3’ |

**Supplementry Table 1b. Transferrin binding protein B amplification and sequencing primers**

| Forward external primer | 5’ CAATCCATTGGTAAATCAG 3’ |
| --- | --- |
| Reverse external primer | 5’ GCCGTCTGAAGCCTTATTC 3’ |
| Forward internal sequencing primer isotype I – 1 | 5’ CTAYAAAGGSARHRAWCCTTCC 3’ |
| Forward internal sequencing primer isotype I – 2 | 5’ CCGATTTYGGKMTGACYAG 3’ |
| Reverse internal sequencing primer isotype I – 1 | 5’ CCRCCTTCCTGATTGGAGG 3’ |
| Reverse internal sequencing primer isotype I – 2 | 5’ CTGAAATGCCGCCTTATTGCC 3’ |
| Forward internal sequencing primer isotype II – 1 | 5’ GACGGYTATATYTTYTATMAMGG 3’ |
| Forward internal sequencing primer isotype II – 2 | 5’ GAAACCAARSAACATCCCTTTG 3’ |
| Reverse internal sequencing primer isotype II – 1 | 5’ GAAGCATTGCCGCTCCAGC 3’ |
| Reverse internal sequencing primer isotype II – 2 | 5’ CTGTTCCGCCGTTTKTACC 3’ |

**Supplementary Table 2: Data collection and refinement statistic for the *Nm* B16B6 TbpB crystal structure solved in this study.**

|  | *Neisseria meningitidis* B16B6 TbpB (isotype I) |
| --- | --- |
| **PDB code** | 4QQ1 |
| **Data collection*** |  |
| Space group | C2 |
| Cell dimensions: |  |
| *a*, *b*, *c* (Å) | 135.33 - 99.97 - 160.15 |
| a, b, c (°) | 90.00 - 93.47 - 90.00 |
| Resolution (Å) | 45.2 - 3.3 (3.44 - 3.33) |
| *I* / σ*I* | 17.5 (4.04) |
| Completeness (%) | 98.7 (97.7) |
| R_sym_ | 0.09 (0.67) |
| **Refinement*** |  |
| Resolution (Å) | 45.2 – 3.33 |
| No. reflections | 31027 |
| *R*_work_ / *R*_free_ | 0.25/0.30 |
| **No. atoms** | 11828 |
| Protein | 11773 |
| Ligands | 25 |
| Water | 30 |
| ***B*-factors** |  |
| Protein | 125.4 |
| Ligands | 156.3 |
| Water | 71.1 |
| **R.m.s deviations** |  |
| Bond lengths (Å) | 0.004 |
| Bond angles (°) | 0.984 |
| **Ramachandran** |  |
| Favored (%) | 86.1 |
| Outlier (%) | 0.2 |

*Highest resolution shell is shown in parenthesis

**Supplementry Table 3.** Strain Characteristics and Accession Numbers for TbpB and TbpA

| **Strain Characteristics** | | | | **Accession Number** | |
| --- | --- | --- | --- | --- | --- |
| **STRAIN** | **SEROGROUP** | **COUNTRY** | **YEAR** | **TbpA** | **TbpB** |
| B16B6 | B | - | - | KP776831 | KP776937 |
| M982 |  | - | - | KP776832 | KP776933 |
| MC58 | B | UK | 1983 | KP776833 | KP776938 |
| K454 | - | - | - | KP776834 |  |
| S3032 | - | - | - | KP776835 | KP776911 |
| M136 | - | - | - | KP776836 | KP776912 |
| M978 | B | USA | - | KP776837 | KP776913 |
| M990 | - | - | - | KP776838 | KP776914 |
| M1080 | - | - | - | KP776839 | KP776915 |
| P3006 | - | - | - | KP776840 | KP776916 |
| P2996 | - | - | - | KP776841 | KP776917 |
| M1011 | - | - | - | KP776842 | KP776918 |
| P2396 | - | - | - | KP776843 | KP776919 |
| 34WRR | - | - | - | KP776844 | KP776920 |
| P3459 | - | - | - | KP776845 | KP776922 |
| B1SR | - | - | - | KP776846 | KP776923 |
| B2RR | - | - | - | KP776847 | KP776924 |
| NMXYZ | - | - | - | KP776848 | KP776925 |
| 3662 | - | CANADA | - | KP776849 | KP776926 |
| FH#18270 | - | - | - | KP776850 | KP776927 |
| NMRUGrpY | - | - | - | KP776851 | KP776928 |
| 3-6 | - | - | - | KP776852 | KP776929 |
| LCDC#88252 | - | - | - | KP776853 | KP776930 |
| LCDC#89169 | - | - | - | KP776854 | KP776931 |
| LCDC#88137 | - | - | - | KP776855 | KP776932 |
| LCDC#89554 | - | - | - | KP776856 |  |
| N16T2K | A | GHANA | - | KP776857 |  |
| 116 | - | - | - | KP776858 | KP776934 |
| H44/76 | - | - | - | KP776859 | KP776935 |
| CE1431 | - | - | - | KP776860 | KP776936 |
| S5611 | A | AUSTRALIA | 1977 | KP776861 | KP776939 |
| 6748 | A | CANADA | 1971 | KP776862 | KP776940 |
| "020" | A | NIGER | 1963 | KP776863 | KP776941 |
| 120M | A | PAKISTAN | 1967 | KP776864 | KP776942 |
| 129E | A | GERMANY | 1964 | KP776865 | KP776943 |
| 254 | A | DJIBOUTI | 1966 | KP776866 | KP776944 |
| IAL2229 | A | BRAZIL | 1976 | KP776867 | KP776945 |
| F6124 | A | CHAD | 1988 | KP776868 | KP776946 |
| "00154" | A | CHINA | 1966 | KP776869 | KP776947 |
| **Strain Characteristics** | | | | **Accession Number** | |
| **STRAIN** | **SEROGROUP** | **COUNTRY** | **YEAR** | **TBPA** | **TBPB** |
| "00153" | A | CHINA | 1966 | KP776870 | KP776948 |
| 11-004 | A | CHINA | 1984 | KP776871 | KP776949 |
| 92001 | A | CHINA | 1992 | KP776872 | KP776950 |
| S4355 | A | DENMARK | 1974 | KP776873 | KP776951 |
| 7891 | A | FINLAND | 1975 | KP776874 | KP776952 |
| 14/1455 | A | RUSSIA | 1970 | KP776875 |  |
| H1964 | A | UK | 1987 | KP776876 | KP776953 |
| "010" | A | BURKINA FASO | 1963 | KP776877 | KP776954 |
| 255 | A | BURKINA FASO | 1966 | KP776878 | KP776955 |
| 243 | A | CAMEROON | 1966 | KP776879 | KP776956 |
| S3131 | A | GHANA | 1973 | KP776880 | KP776958 |
| D8 | A | MALI | 1990 | KP776881 | KP776959 |
| "026" | A | NIGER | 1963 | KP776882 | KP776960 |
| 1014 | A | SUDAN | 1985 | KP776883 | KP776961 |
| A4/M1027 | A | USA | 1937 | KP776884 | KP776962 |
| 79128 | A | CHINA | 1979 | KP776885 | KP776963 |
| 79126 | A | CHINA | 1979 | KP776886 | KP776964 |
| G2136 | B | ENGLAND | 1986 | KP776887 | KP776965 |
| 312901 | C | ENGLAND | 1996 | KP776888 | KP776966 |
| AK22 | B | GREECE | 1992 | KP776889 |  |
| BZ163 | B | HOLLAND | 1979 | KP776890 | KP776967 |
| B6116/77 | B | ICELAND | 1977 | KP776891 |  |
| NZ94/155 | C | NEW ZEALAND | 1994 | KP776892 |  |
| SB25 | C | SOUTH AFRICA | 1990 | KP776893 |  |
| BRAZ10 | C | BRAZIL | 1976 | KP776894 | KP776968 |
| L93/4286 | C | ENGLAND | 1993 | KP776895 |  |
| F1576 | C | GHANA | 1984 | KP776896 |  |
| M597 | C | ISRAEL | 1988 | KP776897 | KP776969 |
| D1 | C | MALI | 1989 | KP776898 | KP776971 |
| NGP20 | B | NORWAY | 1969 | KP776899 | KP776972 |
| 90/18311 | C | SCOTLAND | 1990 | KP776900 | KP776973 |
| MA-5756 | C | SPAIN | 1985 | KP776901 | KP776974 |
| 38V1 | B | USA | 1964 | KP776902 |  |
| BZ83 | B | HOLLAND | 1984 | KP776903 |  |
| NG080 | B | NORWAY | 1981 | KP776904 |  |
| NG144/82 | B | NORWAY | 1982 | KP776905 | KP776977 |
| NGPB24 | B | NORWAY | 1985 | KP776906 |  |
| 196/87 | C | NORWAY | 1987 | KP776907 |  |
| 400 | B | AUSTRIA | 1991 | KP776908 | KP776978 |
| 931905 | B | NETHERLANDS | 1993 | KP776909 | KP776979 |
| NZ91/40 | - | - | - | KP776910 |  |
| **Strain Characteristics** | | | | **Accession Number** | |
| **STRAIN** | **SEROGROUP** | **COUNTRY** | **YEAR** | **TBPA** | **TBPB** |
| M992 | - | - | - |  | KP776921 |
| C751 | A | GAMBIA | 1983 |  | KP776957 |
| 500 | C | ITALY | 1984 |  | KP776970 |
| 8680 | B | CHILE | 1987 |  | KP776975 |
| BZ169 | B | HOLLAND | 1985 |  | KP776976 |
