# Supplementary figures and images for: Patterns of structural and sequence variation within isotype lineages of the Neisseria meningitidis transferrin receptor system
Source: Microbiologyopen. 2015 Mar 19;4(3):491–504. doi: 10.1002/mbo3.254 (PMC4475390; doi:10.1002/mbo3.254)

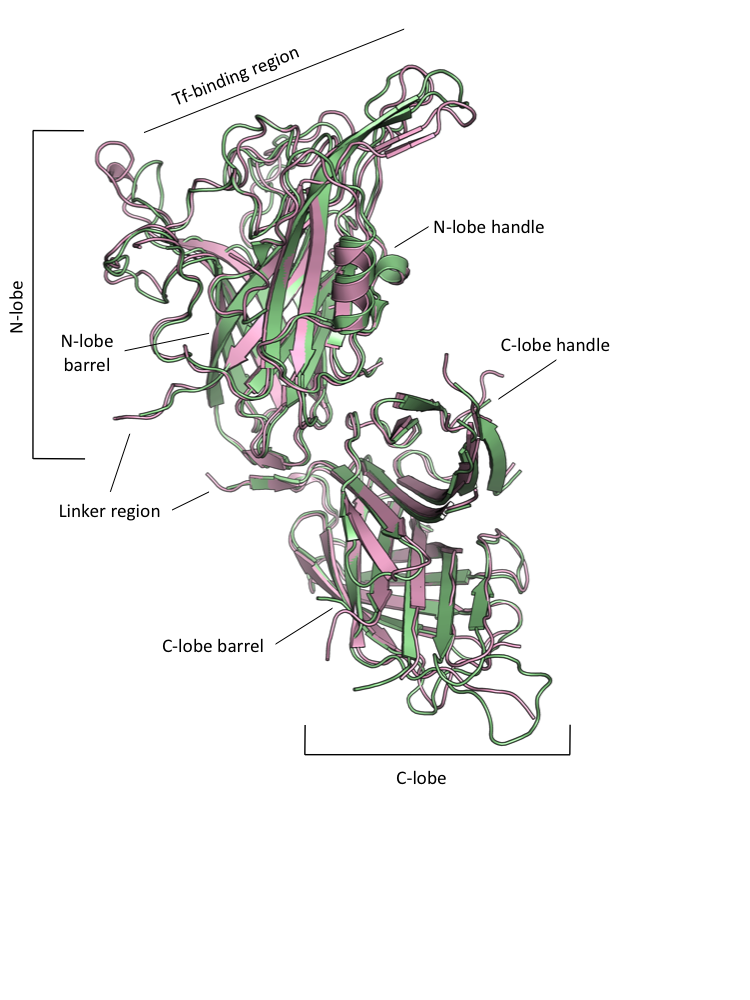

Supplement: Figure S1 — Superimposition of the N. meningitidis strain K454 (pink; PDB ID: 3V8U) and strain M982 (green PDB ID: 3VE2) TbpB crystal structures with key regions identified. [file mbo30004-0491-sd2.tif]

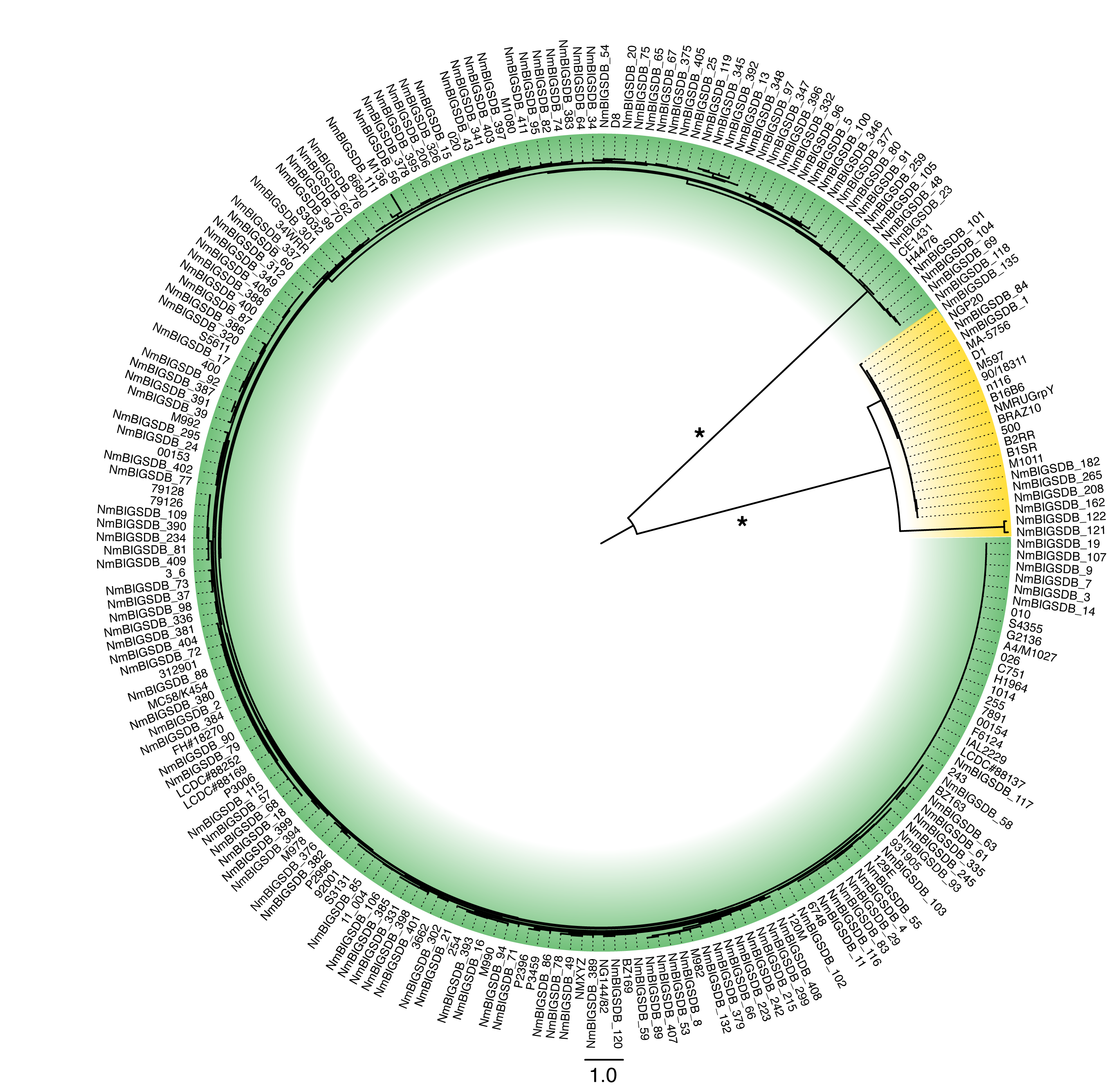

Supplement: Figure S2 — TbpB C lobe phylogenetic tree. TbpB C-lobe sequences from 229 strains were included in this analysis. Two primary clades are identified within this tree corresponding to the isotype I and isotype II tbpB lineages (yellow background and green backgrounds respectively). Support values for primary branches are depicted by a “*” identifying 100% support for these two branches. [file mbo30004-0491-sd3.tif]

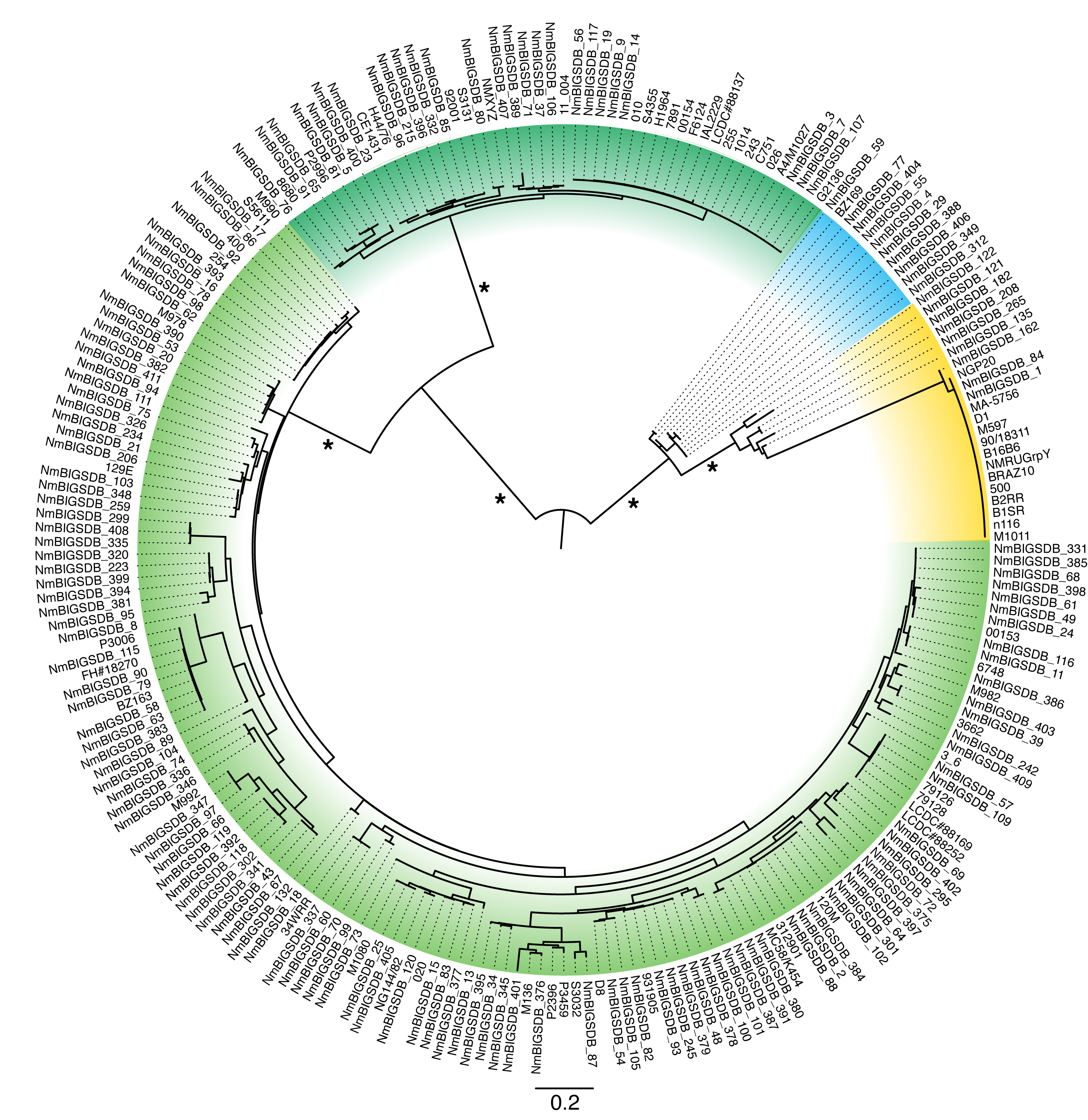

Supplement: Figure S3 — TbpB N lobe phylogenetic tree. TbpB C-lobe sequences from 229 strains were included in this analysis. Two primary clades are identified within this tree corresponding to the isotype I and isotype II tbpB lineages (yellow background and green/blue backgrounds respectively). Support values for primary branches are depicted by a “*” identifying 100% support for these two branches. [file mbo30004-0491-sd4.tif]
